# Digital Rights Management: The Librarian’s Guide

**DOI:** 10.5195/jmla.2018.461

**Published:** 2018-07-01

**Authors:** A. Robin Bowles

**Affiliations:** Nursing and Life Sciences Librarian, Falvey Library, Villanova University, Villanova, PA

Digital rights management (DRM) is an important and inseparable feature of electronic resources and an increasingly important facet of collection development and library services. Understanding the various types of DRM and how they function is an essential element of modern librarians’ toolkits, regardless of their role in their organizations. *Digital Rights Management: The Librarian’s Guide* is an edited volume that offers such an understanding. Authored by an impressive collection of law librarians and copyright experts across the nation and edited by Catherine A. Lemmer, director of Lake Forest Library, and Carla P. Wale, of Temple University Beasley School of Law Library, the volume offers a comprehensive view of how DRM shapes all kinds of library decision-making regarding acquisition, access, and management of electronic resources. It also explores how DRM can both be both a blessing and a curse for the continued evolution of electronic information.

The book’s first chapter, authored by Frederick W. Dingledy and Alex Berrio Matamoros, usefully frames the issues by providing a review of both broad definitions and specific descriptions of DRM systems of the past and in current use. It is followed by an even further in-depth look at some of these systems authored by Jasper Tran. Amanda Watson then zeros in more closely on the crucial matters of digital authentication methods, encryption, and the particularly thorny world of e-book DRM. These chapters not only provide an essential basis for those who work with DRM professionally, but they are also contrasted nicely by the book’s final chapter, by Dana Neacsu, that examines alternatives to current DRM options and discusses alternative models for protecting copyrighted works in a library context.

Brian R. Huffman and Victoria J. Szymczak give detailed suggestions on how to evaluate resources and licenses for digital collections. They focus specifically on rights management and DRM implementations and helpfully discuss organizational management and workflow considerations for libraries looking to unify their practices related to DRM. The sample language that the authors provide is especially helpful and applicable to a broad range of library use cases. This is a gift for those who are looking to make new vendor agreements or just update existing contracts to ensure that they match current use and needs.

Of special interest to medical librarians will be the chapter authored by Roberta F. Studwell and Jordan A. Jefferson, which discusses patron privacy issues with an extensive exploration of how DRM and other electronic systems have complicated protecting the privacy of our patrons, and the authors helpfully propose sample language for both library privacy policies and vendor contracts that align with current privacy laws and American Library Association guidelines. A fascinating chapter by Benjamin J. Keele and Jere D. Odell regarding how DRM systems can be useful to those publishing, hosting, or authoring open access works is also included. Special attention is paid here to the role that librarians play in advising potential open access authors on technical, copyright, and licensing matters.

The previously mentioned chapter by Keele and Odell and a chapter by Renate L. Chancellor and Heather A. Wiggins, respectively, discuss matters related to the use of DRM in the context of copyright laws and protected uses of copyrighted works. The first offers a useful perspective on how DRM protections can be a positive force in protecting open access works and guides librarians who advise authors who are unsure about how to go about protecting their rights when they publish under open access models. The second examines how DRM practices work within the legal definitions of copyright and other protections for authors and publishers, but then how these same systems expand control beyond the strict legal protections. The chapter highlights the wide expanse between how US copyright laws are written and how DRM is used by publishers to enforce those protections at the expense of the rights of readers and libraries.

If the book has a weak point, it stems from the interdisciplinary nature of the subject matter at hand. To understand DRM applications, it is necessary to take a multifaceted approach that includes the legal aspects of copyright, technical aspects surrounding digital access, and library management issues of user access concerns. The arrangement of the chapters has the reader swinging wildly among these foci without a progressive level of complexity between chapters. The text itself tacitly acknowledges this, relating how evaluating and managing DRM-protected resources can overwhelm even experienced library professionals. The chapter dedicated to how to arrange or modify library management structures and workflow regarding electronic resources and their DRM explicitly suggests that acquisitions, access, and technology teams should work closely together or be merged into combined departments to ensure that DRM issues are handed holistically across library functions.

*Digital Rights Management: The Librarian’s Guide* provides an excellent starting point for librarians at any level who are seeking to understand the current range of DRM systems, their functional mechanics of access and copy protections, and how the various types of DRM and their restrictions play into larger considerations for collection development, electronic acquisitions processes, and user services. As electronic resources continue their rise in dominating library budgets and collections, this timely book provides a convenient means for librarians, administration, and staff to understand how DRM will shape the future of library collections and the broad use of information in our age.

